# Basin uplift and related climate dynamics drive far-field submarine landslides

**DOI:** 10.1093/nsr/nwag342

**Published:** 2026-06-04

**Authors:** Qiliang Sun, Vittorio Maselli, Xingxing Wang, Shucheng Xie

**Affiliations:** State Key Laboratory of Geomicrobiology and Environmental Changes, Hubei Key Laboratory of Marine Geological Resources, China University of Geosciences, Wuhan 430074, China; Laboratory for Marine Mineral Resources, Qingdao National Laboratory for Marine Science and Technology, Qingdao 266061, China; Department of Chemical and Geological Sciences, University of Modena and Reggio Emilia, Modena 41121, Italy; State Key Laboratory of Geomicrobiology and Environmental Changes, Hubei Key Laboratory of Marine Geological Resources, China University of Geosciences, Wuhan 430074, China; State Key Laboratory of Geomicrobiology and Environmental Changes, Hubei Key Laboratory of Marine Geological Resources, China University of Geosciences, Wuhan 430074, China

**Keywords:** slope instability, basin uplift, climate dynamics, mass-transport deposits, Southeast Asia

## Abstract

Submarine landslides can generate catastrophic cascading hazards, including tsunamis. Tectonics and climate have long been hypothesized to influence slope instability, but how they control repeated slope failures over geological time scales is still unclear. In this study from the South China Sea, we find that >90% of the ∼1900-m-thick Plio-Quaternary succession consists of repeated submarine landslide deposits, and they commenced suddenly at about 5.5 Ma. We demonstrate that uplift of the Red River basin and associated strengthening of the Indian Summer Monsoon drive a regime shift in sediment supply, characterized by a sustained increase that persists to the present, steepening the margin slope and promoting sediment overpressure, thereby triggering widespread mass wasting. This study demonstrates a teleconnection between continental tectonic–climatic changes and submarine landslide activity, thereby advancing our understanding of repeated slope-instability phenomena within a source-to-sink stratigraphic framework and contributing to the reconstruction of Earth’s history.

## INTRODUCTION

Submarine slope failures are common along continental margins globally [1–4]. Compared to their subaerial counterparts, they have greater volumes, up to 10^5^ km^3^ [3,5], and the potential to generate damaging tsunamis, which can impact coastal communities [6]. They also pose a risk to marine infrastructure, such as telecommunication cables, which carry over 99% of global internet traffic [7]. Consequently, numerous studies worldwide have focused on understanding the timing and mechanisms of submarine landslide emplacement at or near the modern seafloor—likely occurring during the Late Pleistocene to Holocene—to assess the potential for future events in a given region. Far fewer studies have documented repeated landslides over a longer time scale spanning several millions of years, let alone reveal their origins.

Submarine landslides could be preconditioned by many factors, such as sea-level/climate change, gas hydrate dissociation, overpressure, and high sedimentation rates. Repeated slope failures on glaciated margins facing the North Atlantic Ocean have been well studied (see for example the Storegga Slide complex on the mid-Norwegian margin [8]), and they have been hypothesized to be a consequence of the control of glacial-interglacial cycles over sediment caliber and supply rate and earthquakes related to isostatic rebound [2]. Repeated submarine landslides are also commonly observed in non-glaciated regions, especially in those margins supplied by large rivers with high sediment yield [9]. In such settings, long-term fluctuations in sediment supply to continental slopes are primarily driven by the environmental changes within the drainage basin, such as surface uplift and climate variations [10]. By modifying river drainage systems and regional climate patterns, and by increasing weathering and topographic gradient, basin tectonics may boost sediment production and transport to the adjacent offshore sedimentary basins, potentially overriding the effect of sea-level changes [4,11]. Previous studies on submarine landslides primarily focused on understanding the preconditioning and short-term triggering mechanisms occurring in the marine environment [1–3,12]. In contrast, far fewer studies have examined how onshore tectonics and associated climate feedbacks may influence submarine landslides in adjacent marine basins, here considered a far-field domain relative to the area where uplift is occurring.

South and East Asia are representative non-glaciated regions characterized by large river catchments such as the Indus, Ganges-Brahmaputra, Mekong, Red, Pearl, and Yangtze Rivers (Fig. S1). Sediment production and transport to the Indian Ocean and South China Sea (SCS) are modulated by the precipitation dynamics due to the Indian Summer and East Asian monsoons and the erosional processes occurring in the high-elevation regions of the Himalaya and Tibetan Plateau [13]. Sediment delivery from these rivers to the ocean has resulted in the accumulation of >10-km-thick clastic sequences in ∼10–25 million of years [14,15], where large-scale submarine landslides and associated mass-transport deposits (MTDs) have been discovered [16]. Consequently, these basins are key sites to study how variations in basin climate and topography over long-term time scales may influence submarine landslides in non-glaciated regions [3,16,17].

**Figure 1.**
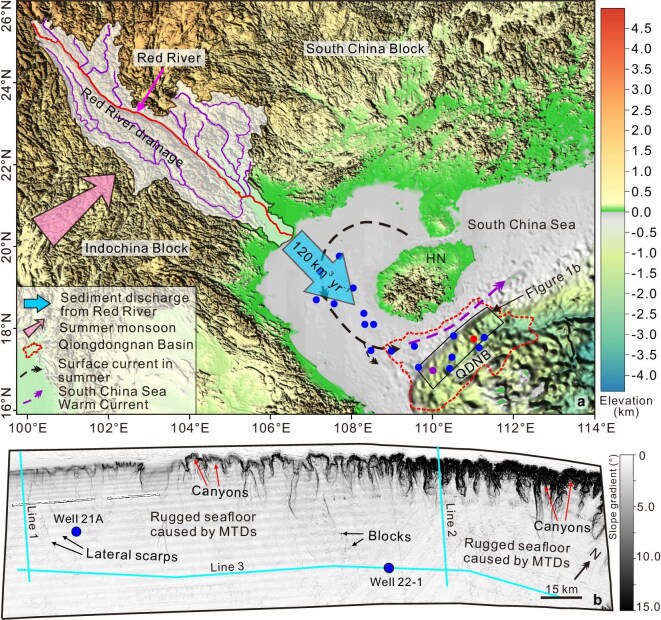
(a) Geological setting of the study area, with the Red River marked by a red solid line and the main tributaries in purple. The black dashed line is the summer surface circulation in the SCS while the purple dashed line is SCS Warm Current (modified from [20]). The red dashed line outlines the Qiongdongnan Basin (QDNB); HN: Hainan Island. Note that the Red River Fault Zone (RRFZ) overlaps with the Red River (red solid line). The sediment discharge of the modern Red River is from [25]. The pink arrow shows the direction of the Indian Summer Monsoon (modified from [47]). The black rectangle marks the locations of panel (b). The boreholes used to analyze the sediment sources by [19] are the blue dots, while the purple dot is well 21A and the red dot is Well 22-1. (b) Slope gradient map showing canyons, slope failure scarps and landslide deposits (rugged seafloor) in the study area. Locations of Figs 2 (Line 1), 3 (Line 2), 4 (Line 3), Well 21A, and Well 22-1 are labelled.

Using geophysical and borehole data, we identified over 500 MTDs in the Pliocene and Quaternary succession of the northwestern SCS offshore the Red River (Fig. 1). Such a large number of landslide deposits accumulated over approximately 5 million years has not been previously documented. This study investigates how climate (particularly monsoonal variability) and tectonic processes occurring in the catchment area may control far-field, repeated slope failures in the SCS, providing new insights into source-to-sink sediment routing systems.

## GEOLOGICAL SETTINGS

**Figure 2.**
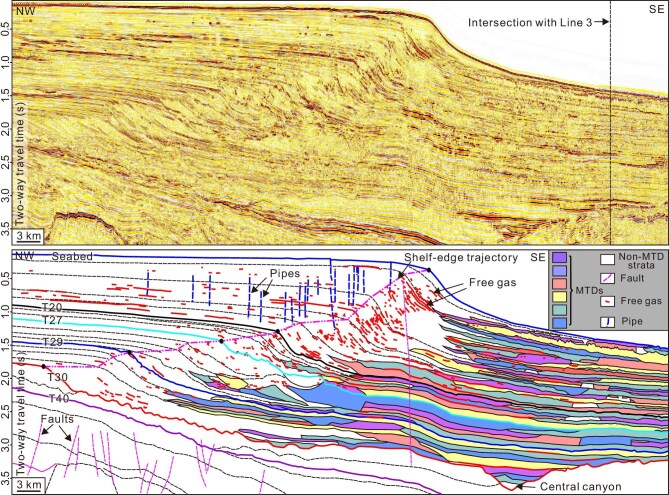
Seismic profile (Line 1) in the western Qiongdongnan Basin and its interpretation. The sequence boundaries (T40, T30, T29, T27, T20, and seabed), free gas (red short solid lines), vertical fluid escape pipes (blue dashed lines), faults (pink dashed lines), central canyon and mass-transport deposits (MTDs; colored polygons) are labelled. Repeated MTDs are stacked and occur after surface T30. See location in Fig. 1b.

**Figure 3.**
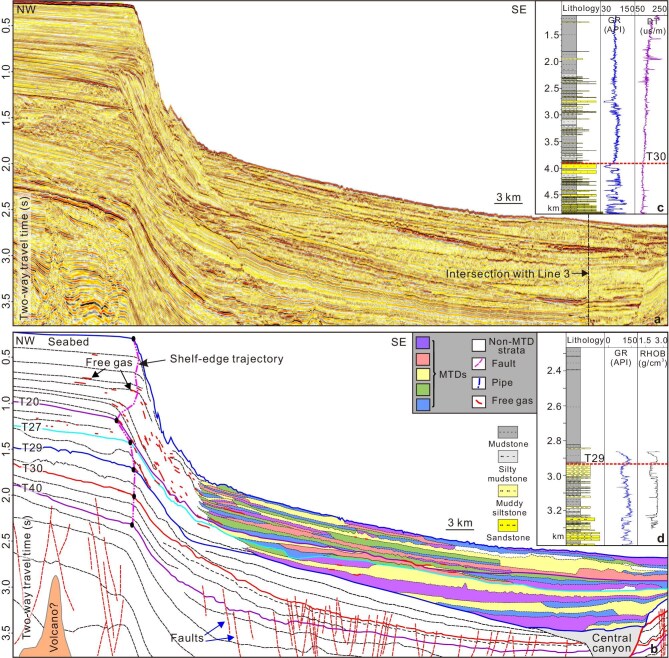
Seismic profile (Line 2) in the eastern Qiongdongnan Basin (a) and its interpretation (b). See location in Fig. 1b. The sequence boundaries (T40, T30, T29, T27, T20 and seabed), free gas (red short solid lines), faults (red dashed lines), Central canyon (grey polygon) and mass-transport deposits (MTDs; colored polygons) are labelled. Repeated MTDs are stacked and immediately occur after surface T29. The variances of lithology in the western Qiongdongnan Basin (Well 21A; Fig. 3c) and in the eastern Qiongdongnan Basin (Well 22-1; Fig. 3d) are shown.

**Figure 4.**
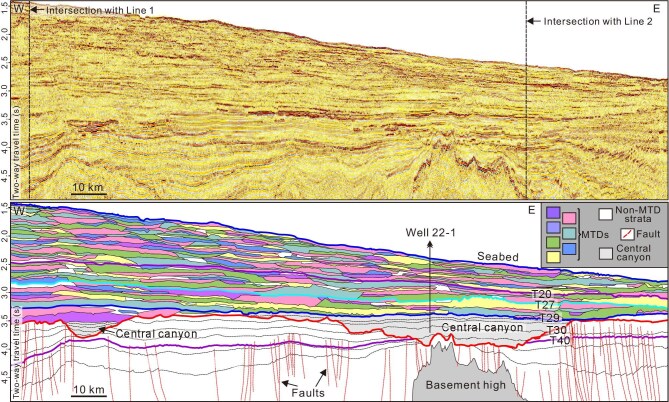
Seismic profile (Line 3) along the middle of deep-water region of Qiongdongnan Basin (central canyon) and its interpretation. See location in Fig. 1b. The sequence boundaries (T40, T30, T29, T27, T20 and seabed), free gas (red short solid lines), faults (red dashed lines), central canyon (grey polygon) and mass-transport deposits (MTDs; colored polygons) are labelled. Note that the onset of repeated MTDs becomes younger and the MTDs thin towards the east.

The study area is located in the Qiongdongnan Basin (QDNB), northwestern SCS (Fig. 1 and Figs S2 and S3), a Cenozoic basin covering an area of 80 000 km^2^. The QDNB experienced an Eocene-Oligocene rifting stage and a Miocene-present post-rifting/subsidence stage (Fig. S4; [18]). The stratigraphic architecture of the QDNB is well constrained, largely owing to intensive hydrocarbon exploration over the past five decades (Fig. S4). In this study, we subdivide the stratigraphy using five regionally extensive seismic horizons (T40 at ∼10.5 Ma, T30 at ∼5.5 Ma, T29 at ∼4.2 Ma, T27 at ∼2.7 Ma, and T20 at ∼1.8 Ma; Figs 2–4 and Fig. S4) that have been previously identified and dated using biostratigraphy and zircon U–Pb geochronology [18–22]. Since the Pliocene (horizon T30), up to ∼1.9 km of predominantly fine-grained sediments, largely sourced from the Red River drainage basin [19,23], have accumulated in the QDNB, overlying sand-rich sequences associated with Miocene submarine fans [22]. The Red River basin, which covers an area of 13.9 × 10^4^ km^2^, has undergone episodic surface uplift (1400–1500 m) and pronounced river incision (∼1400 m) since the Pliocene [11], driven by the India-Eurasia collision [24]. At present, the Red River delivers ∼120 km^3^/yr of sediments into the SCS [25], making it the largest sediment source in the northwestern part of the basin [26].

South and East Asia are strongly influenced by the Indian Summer Monsoon and the East Asian Monsoon, both of which intensified after the late Miocene in response to global climate change [27]. Moreover, the Indian Summer Monsoon was further strengthened by surface uplift of the Red River drainage basin [11]. At the same time, wind-driven surface currents, including west-to-east-flowing warm current, developed in the northwestern SCS [20], facilitating sediment transport along the QDNB shelf and slope [21]. MTDs in the QDNB have been described by a number of studies, however they mainly focused on the Central Canyon in the deep-water region of the basin or at the modern seafloor [18,20,28].

## RESULTS

### Stratigraphic architecture of the QDNB

**Figure 5.**
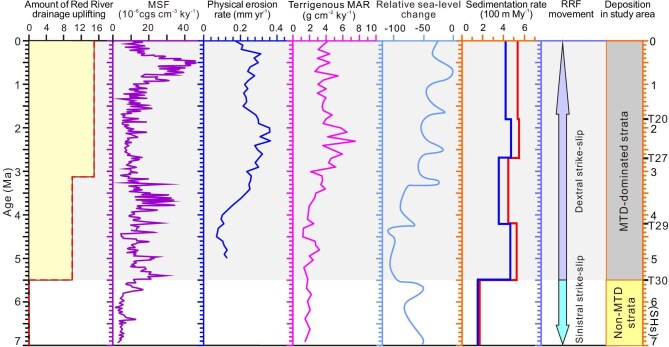
Tectonic and climatic factors in/around the study area. The episodic uplifting (two stages; total amount of uplifting: ∼1500 m) of the Red River drainage since the onset of Pliocene is inferred from [11]. The magnetic susceptibility flux (MSF) is from ODP Site 758, which is a proxy for the strength of the summer monsoon [10]. The increase of erosion rates [48] and high terrigenous mass accumulation rate (MAR; [27]) reflect surface uplift and monsoon strengthening. The relative sea-level change of Qiongdongnan Basin (QDNB) is from [49]. The direction of the Red River strike-slip Fault (RRF) is from [38]. Sedimentation rates in the western QDNB (red solid line) and eastern QDNB (blue solid line) are calculated in this study. The frequent slope failures (MTDs) coincide with the surface uplifting, monsoon strengthening, increased erosions and sedimentation rates. SHs: seismic horizons.

Seismic reflections from pre-T30 strata dip seawards ∼1.1° in the western part of QDNB and become steeper to ∼6.2° after T30 (∼5.5 Ma) (Fig. 2). The shelf-edge trajectory, defined as the pathway taken by the shelf-edge position through time, has prograded seaward ∼82 km since T30 in the western part of QDNB (Fig. 2 and Fig. S2). In the eastern part of QDNB, the shelf-edge has mainly aggraded since T30, with short-lived periods of landward migration (Fig. 3 and Fig. S2). Sedimentation rates in the western QDNB are ∼173 m/Ma (T40–T30), ∼541 m/Ma (T30–T29), ∼454 m/Ma (T29–T27), ∼561 m/Ma (T27–T20), and ∼546 m/Ma (T20–seafloor), while they are ∼153 m/Ma (T40–T30), ∼472 m/Ma (T30–T29), ∼360 m/Ma (T29–T27), ∼479 m/Ma (T27–T20), and ∼426 m/Ma (T20 to seafloor) in the eastern QDNB (file S1). Over the same time interval, the strata thickness in the western part is much larger than that in the eastern part (Fig. 4), which indicates that sedimentation rates in the western QDNB are consistently higher than those in the eastern QDNB. On average, sedimentation rates across the slope increased approximately threefold from pre- to post-T30, rising from ∼160 to ∼500 m/Ma (Fig. 5).

In addition to the marked increase in sedimentation rates since the onset of the Pliocene, lithology also changes across surface T30 (western QDNB; Fig. 3C) and T29 (eastern QDNB; Fig. 3D). In the western QDNB, pre-T30 strata consist mainly of thick-bedded sandstones and siltstones, whereas above T30 they are dominated by mudstones often intercalated with thin-bedded muddy siltstones (Fig. 3C). In the eastern QDNB, the pre-T29 strata are dominated by muddy siltstones and sandstones, whereas the overlying succession consists primarily of mudstones with only sparse, thin beds of muddy siltstone (Fig. 3D). Moreover, sediment grain size generally fines from west to east across the QDNB, and a similar fining trend is observed from older to younger strata (Fig. 3C and D).

### Nature and timing of slope deposits

Two main types of seismic facies are recognized: Type I is characterized by parallel/layered and high amplitude seismic reflections, laterally continuous and often wavy, which we interpreted as turbidites, contourites and hemipelagites [18,20]; Type II is characterized by chaotic/transparent seismic facies (Figs 2–4 and Figs S5 and S6), often with contorted reflections, which we interpreted as MTDs [5]. No MTDs (Type II seismic facies) are observed before T30 (5.5 Ma), while repeated MTDs begin to occur above T30 in the western QDNB or above T29 in the eastern QDNB (Fig. 2).

Across the entire basin, we identified 517 MTDs whose boundaries are delineated by continuous, high-amplitude, negative-polarity seismic reflections (Figs 2–4). The actual number of MTDs in the study area may be significantly higher, as smaller deposits below seismic resolution, as well as amalgamated or stacked MTDs, or MTDs eroded by younger events (Figs 2–4), remain undetected. Remarkably, MTDs account for over 90% of the Pliocene and Quaternary strata in the deep-water regions of the QDNB (Fig. S7). The Plio-Quaternary gradually thins northeastward from 1.89 km (2.1 s TWT) in the western QDNB to 0.45 km (0.5 s TWT) in the eastern QDNB (Fig. 4). Submarine landslides’ scarps can be clearly observed at the present-day shelf edge, and some scarps are also observed on the slopes (Fig. S3). Moreover, canyons are also well developed in the study area, especially in the eastern SCS, and landslides’ scarps often delineate their flanks [28].

Enhanced negative-polarity seismic anomalies with high root mean square (RMS) attribute amplitude (Fig. S5), with sizes from less than 1 km to about 10 km and associated with acoustic blanking, are widespread, particularly on the shelf of the western QDNB (Fig. 2) and are interpreted as gas-charged sediments [29]. They are also associated with narrow pipe-like structures with widths of 100 s m which may represent fluid pathways (Fig. 2).

Faults with clear offsets are largely confined to pre-T30 sediments (Figs 2–4); only a few extend to the seabed near the slope (Fig. 2).

## DISCUSSION

### Sudden onset of repeated slope failures sustained over a period of 5.5 Myr

Repeated submarine slope failures are common along many continental margins and island flanks [30]. However, they are typically characterized by a few events occurring over hundreds of thousands of years or less [2]. In the study area, while no MTDs have been observed before horizon T30 (∼5.5 Ma), at least 517 MTDs, representing >90% of the 1900-m-thick Plio-Quaternary (post-T30) stratigraphic succession, have been identified (Fig. 4). To the best of our knowledge, such a long (∼5.5 Myr) record and large number (>517 events) of repeated landslides have not been documented before in any other margins worldwide.

Different mechanisms may trigger submarine slope failures, and earthquakes are often invoked as an important one [5]. However, earthquakes are unlikely to have been the primary driver of the post-T30 slope failures in the QDNB. Since at least the late Miocene, the basin has remained in a tectonically quiescent phase dominated by thermal subsidence [20], as indicated by the absence of major faults cutting the Plio-Quaternary strata (Figs 2–4 and Fig. S5). Consequently, tectonic activity cannot readily account for the abrupt onset of widespread slope failures after T30. Furthermore, although the Red River fault system may occasionally generate earthquakes—and thus cannot be entirely excluded as a sporadic trigger of some post-T30 slope instability events—it is located onshore more than 400 km from the study area (Fig. S1), making a significant influence on slope instability in the QDNB, particularly in the more distant eastern basin, unlikely.

Gas hydrates, which may be responsible for increasing pore pressure when they dissociate [31], have also been reported in the QDNB, however, only in the lower slope to abyssal plain regions [18], far away from landslide headscarps. Therefore, also gas hydrate dissociation cannot fully explain the occurrences of repeated slope failures. Sea-level changes may also influence slope instability, although this link has not been proven statistically [32]. The limited chronological constraints available for the MTD-rich intervals in the study area prevent us from assessing potential links between MTD occurrence and Milankovitch-scale sea-level oscillations. In addition, Fig. 5 shows that the increase in sediment supply—reflected by higher physical erosion rates and terrigenous mass accumulation rates—across and after T30 (western QDNB) and T29 (eastern QDNB) occurred during a phase of gradual sea-level rise. This pattern contrasts with classical sequence stratigraphic models, which typically predict enhanced sediment delivery during lowstand conditions. The apparent decoupling between sea-level fluctuations and sedimentation rates therefore suggests that sea-level change was unlikely to have been the primary control on the enhanced sediment supply in the basin.

**Figure 6.**
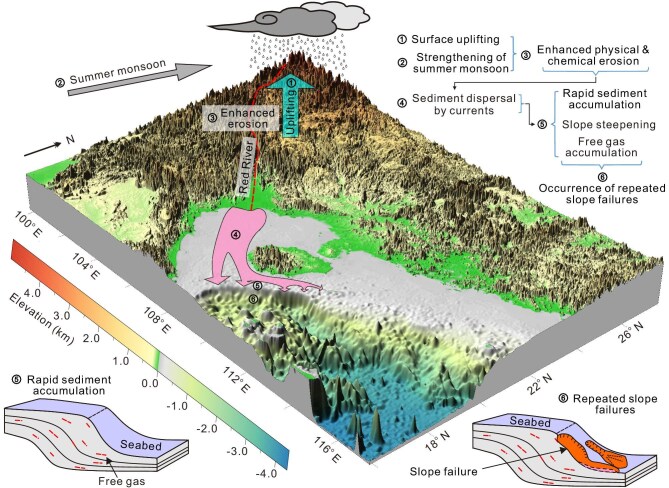
Model for basin uplift and related climate dynamics driving far-field submarine landslides.

The onset of slope failures between 5.5 Ma (T30, western QDNB) and 4.2 Ma (T29, eastern QDNB) coincides with a threefold increase in sediment accumulation rates (Fig. 5), which led to a basin-wide shelf and slope aggradation, as well as significant progradation in the western QDNB (Figs 2 and 3). The shift in sediment accumulation is also accompanied by a pronounced lithological change, from predominantly coarse-grained facies to mud-rich successions across horizons T30 and T29 (Fig. 3C and D). High sedimentation rates are widely recognized as an important preconditioning factor for submarine slope failures [2,9], as increased sediment supply can steepen the slope and thereby reduce its stability [33]. In the QDNB, for instance, slope gradient in the progradational western QDNB increased from ∼1° to ∼6° between surfaces T40 and T29 (Figs 2 and 6), making slope sediments more susceptible to failure. Rapid sediment accumulation may also lead to undercompaction of fine-grained deposits [1]; Pore-pressure simulations (file S2) indicate that overpressure quickly builds up below 50 mbsl when average sedimentation rate is greater than 500 m/Myr. In addition, rapid burial of fine-grained sediments promotes the trapping of methane from thermal or biogenic origin, which reduces sediment shear strength [34,35]. The presence of widespread seismic anomalies, often associated with vertical pipe-like discontinuities, indicates that large amounts of free gas occur throughout the basin (Fig. 2 and Fig. S8). Taken together, these lines of evidence suggest that gas-charged, undercompacted muddy sediments, combined with steeper slopes, likely contributed to the repeated slope failures that affected the margin over the past 5.5 Myr.

### What drives higher sediment supply to the basin?

Geochemical analyses, including U-Pb dating and Hf isotope of zircons, Rare Earth Elements composition and mineral assemblages from borehole samples indicate that the Red River drainage basin has been the dominant sediment source for the QDNB since at least the Late Miocene [23]. Consequently, any tectonic and/or climatic changes within this river basin could have influenced sedimentation rates in the QDNB. We propose that the sharp increase in sedimentation rates observed in the study area since the Pliocene (Fig. 6), which has been described also for the adjacent Yinggehai Basin [36], is likely related to the rapid uplift of the Red River basin resulting from the India-Eurasia collision [11,24,37]. Analysis of topographic data and longitudinal profiles of the tributaries of the Red River revealed that the basin experienced 1400–1500 m of surface uplift, which drove ∼1400 m incision of the paleo-Red River [11]. Topographic uplift also intensified the Summer Monsoon and associated rainfall [38,39], which increased chemical weathering as indicated by the increase of fine-grained sediments delivery to the basin ([19]; Fig. 3C and D) and associated magnetic susceptibility fluxes ([39]; Fig. 5). By comparing present-day sediment discharge of the Red River (>120 km^3^/yr) with the sediment volume accumulated within Quaternary basins of some of South Asia’s largest rivers, Métivier and Gaudemer [40] concluded that continental denudation was primarily driven by tectonics and that it has remained constant over the Quaternary, in agreement with our interpretations. Therefore, intense erosion of high-elevation regions in the Red River drainage basin, driven by a strong Indian summer monsoon [11,27,39], generated large volumes of sediment, as indicated by Plio-Quaternary mass budget calculations [40]. This sustained sediment supply led to prolonged high accumulation rates in the QDNB (Fig. 5), promoting slope progradation and oversteepening, as observed in seismic profiles (Figs 2 and 3). It is also likely that tectonic activity along the Red River fault system at the onset of the Pliocene [11] may have further enhanced sediment delivery to the SCS and reduced the time lag between landscape erosion and offshore sediment transport to below the temporal resolution of this study (∼10^6^ yr).

### Implications

This study demonstrates how tectonic–climatic changes occurring within the catchment basin can exert a strong control on far-field landslide activity in the offshore domain. The collision between the Indian and the Eurasian plates gave rise to the elevated regions of Himalaya and Tibet, deeply influencing the evolution and hydrological cycle of the Asian monsoon [10,39]. The rising Himalayan topography has acted as a mechanical barrier to the cold, dry continental air of the Winter Monsoon, limiting its penetration into the region and enhancing the influence of the warm, moist Summer Monsoon from the Indian Ocean and the SCS [39]. Tectonic uplift and associated climate reorganization resulted in increased sediment yield from major rivers originating in these regions, today estimated at 630 km^3^/yr for the Brahmaputra, 490 km^3^/yr for the Ganges, and 550 km^3^/yr for the Mekong, far exceeding the 120 km^3^/yr delivered by the Red River [25]. Seismic studies in the Bengal Bay and the Arabian Sea reveal the presence of large-scale, repeated slope failures [15,17], similar to those observed in the SCS, supporting the idea of a teleconnection between subaerial tectonic-climatic changes and offshore landslides (Fig. 6).

Slope instability is widespread along both glaciated and non-glaciated continental margins, and high sedimentation rates with associated excess pore pressure often served as key preconditioning factors [15,32,35]. However, the processes controlling sediment supply differ markedly between these settings. In glaciated margins, sedimentation and related slope instability are primarily governed by glacial-interglacial cycles [35]. During interglacials, enhanced bottom-current activity promotes the deposition of fine-grained contourites with low shear strength [41], which may be rapidly buried during subsequent glacial periods by large volumes of poorly sorted glaciogenic sediments [42]. In such environments, contourites often act as weak layers that precondition slopes for failure [35]. In addition, gas hydrates that accumulate within these fine-grained deposits on the upper slope during glacial periods may dissociate during warmer interglacials, leading to overpressure and potentially triggering slope instability [43].

Slope failures along non-glaciated margins, particularly those offshore major river systems, are more strongly influenced by environmental changes within river catchments driven by tectonic activity and its effects on regional climate. Tectonic uplift enhances physical/chemical weathering and increases topographic gradients, boosting sediment production and delivery, often overriding the influence of sea-level fluctuations [4,11]. In tropical regions, intense weathering combined with long-distance sediment transport by large rivers favors the supply of fine-grained sediments, as observed in this study (Fig. 3C and D). Mud-rich, rapidly accumulated sequences promote fluid trapping, overpressure development, and ultimately slope instability. Although contourites and gas hydrates also occur along non-glaciated margins, they are typically restricted to deep-water settings [44], far from upper-slope regions where large submarine landslides commonly initiate. Consequently, they likely play a much less significant role in slope instability than their counterparts on glaciated margins. Moreover, variations in sediment discharge and ocean productivity, partly controlled by sea-level changes, may influence weak-layer formation and further contribute to slope instability in low-latitude settings [12,45,46].

The lack of precise dating of landslide deposits within the Plio-Quaternary succession of the QDNB currently prevents establishing a robust link between sediment failure and glacial-interglacial cycles. Future drilling and direct sampling of MTD and non-MTD strata, combined with improved chronological constraints, would help validate seismic interpretations and enable assessment of the frequency of MTD occurrence in relation to glacial-interglacial variability.

## CONCLUSIONS

Submarine landslide deposits constitute >90% of the Plio-Quaternary succession of the Qiongdongnan Basin in the northwestern South China Sea. We show that the repeated occurrence of landslides over the past 5.5 Myr was primarily controlled by the rapid uplift of the Red River drainage basin and associated strengthening of the Indian Summer Monsoon, that boosted sediment production and delivery towards the South China Sea. High rates of sediment accumulation, locally exceeding 500 m/Myr, increased slope gradient and pore pressure, as indicated by abundant free gas, thereby precondition sediment for failure. This study highlights how tectonic-climatic processes in large river catchments can exert a first-order control on slope instability in far-field ocean basins, particularly in tropical regions. This contrasts with glaciated margins, where submarine landslides are more strongly influenced by glacial-interglacial cycles.

## MATERIALS AND METHODS

This study was performed analyzing a combination of geophysical and borehole data. The seafloor was investigated using multibeam bathymetric data covering an area of ∼11 120 km^2^ (Fig. 1B), which were acquired between 2008 and 2012 using SeaBeam2112 and EM122 multibeam systems and processed for navigation, sound-speed corrections, and noise elimination using the CARIS Hips and SIPS 8.1 software. The data have ∼100 m lateral resolution (cell size), which can provide sufficient detail for the discrimination of geomorphological features on the seafloor (Fig. S3), and a vertical resolution between ∼0.6 and ∼6.6 m (3‰ of the water depth) in water depths ranging from ∼200 to ∼2200 m.

We also used post-stacked 3D seismic reflection data comprising nine surveys over an area of ∼19 200 km^2^ (Fig. 1 and Fig. S2), which partially overlaps with the multibeam bathymetry. These seismic data, acquired by the China National Offshore Oil Corporation with a sample rate of ∼4 ms and bin spacing of 12.5 m × 25 m, nearly cover the entire slope and deep-water regions of the QDNB, allowing us to explore MTDs variability throughout the whole basin (Figs 2–4 and Figs S5 and S6). The data are zero-phase and the seismic arbitrary lines extracted from the 3D volume and presented in this study are displayed with SEG (Society of Exploration Geophysicists) normal polarity, whereby a downward increase in acoustic impedance corresponds to a positive reflection event (red on seismic profiles). The dominant frequency is ∼40–45 Hz for the investigated stratigraphic interval. Considering an average sediment velocity of ∼1800 m/s as derived from Well 22-1, the vertical resolution of the seismic data varies from 10 to 12 m.

The seismic horizons (T40, T30, T29, T27 and T20; Figs 2–4 and Fig. S4) are easily correlatable in the shelf and upper slope regions of the QDNB; the correlation becomes more challenging in deeper waters, where the sediments accumulate primarily as landslide deposits, and thus erosion and deformation are common (Figs 2–4 and Fig. S5), as described below. The sedimentation rates (Fig. 5) are calculated from the underformed strata around the shelf-edge trajectories in the western (pink dashed line in Fig. 2) and eastern (pink dashed line in Fig. 3) QDNB.

The lithological and physical properties of the investigated stratigraphic interval were derived from Well 21A and Well 22-1 (Fig. 3C and D). Well 22-1 was drilled from the seabed down to a depth of ∼1168 m, terminating within the deposits of the Central Canyon, whereas Well 21A extends to a depth of ∼4564 m, and fully penetrates the Central Canyon [22]. Lithology and wireline logs [Gamma Ray (GR) and Delta Time (DT) for Well 21A; GR and Bulk Density (RHOB) for Well 22-1] enabled us to investigate the stratigraphic changes occurring across horizon T30.

## Supplementary Material

nwag342_Supplemental_File

## References

[1] Bryn P, Berg K, Forsberg CF et al. Explaining the storegga slide. Mar Pet Geol 2005; 22: 11–9. 10.1016/j.marpetgeo.2004.12.003

[2] Safronova PA, Laberg JS, Andreassen K et al. Late Pliocene-early Pleistocene deep-sea basin sedimentation at high-latitudes: mega-scale submarine slides of the north-western Barents Sea margin prior to the shelf-edge glaciations. Basin Res 2017; 29: 537–55. 10.1111/bre.12161

[3] Sun QL, Alves TM, Lu XY et al. True volumes of slope failure estimated from a quaternary mass-transport deposit in the northern South China Sea. Geophys Res Lett 2018; 45: 2642–51. 10.1002/2017GL076484

[4] Maselli V, Iacopini D, Ebinger CJ et al. Large-scale mass wasting in the western Indian Ocean constrains onset of East African rifting. Nat Commun 2020; 11: 3456. 10.1038/s41467-020-17267-5 32651391 PMC7351987

[5] Moscardelli L, Wood L, Mann P. Mass-transport complexes and associated processes in the offshore area of Trinidad and Venezuela. AAPG Bull 2006; 90: 1059–88. 10.1306/02210605052

[6] Hornbach MJ, Braudy N, Briggs RW et al. High tsunami frequency as a result of combined strike-slip faulting and coastal landslides. Nat Geosci 2010; 3: 783–8. 10.1038/ngeo975

[7] Pope EL, Talling PJ, Carter L. Which earthquakes trigger damaging submarine mass movements: insights from a global record of submarine cable breaks? Mar Geol 2017; 384: 131–46. 10.1016/j.margeo.2016.01.009

[8] Karstens J, Haflidason H, Berndt C et al. Revised Storegga Slide reconstruction reveals two major submarine landslides 12,000 years apart. Commun Earth Environ 2023; 4: 55. 10.1038/s43247-023-00710-y

[9] Piper DJW, Pirmez C, Manley PL. Mass-transport deposits of the Amazon Fan. In: Flood RD, Piper DJW, Klaus A (eds.). Proceedings of the Ocean Drilling Program, Scientific Results, Ocean Drilling Program, College Station, 1997, 109–44.

[10] Prell WL, Kutzbach JE. The impact of Tibet-Himalayan elevation on the sensitivity of the monsoon climate system to changes in solar radiation. In: Ruddiman WF (eds.). Tectonic Uplift and Climate Change. Boston: Springer, 1997, 171–201. 10.1007/978-1-4615-5935-1

[11] Schoenbohm LM, Whipple KX, Burchfiel BC et al. Geomorphic constraints on surface uplift, exhumation, and plateau growth in the Red River region, Yunnan Province, China. Geol Soc Am Bull 2004; 116: 895–909. 10.1130/B25364.1

[12] Wang X, Maselli V, Flessati L et al. Preconditioning of sediment failure by astronomically paced weak-layer deposition. Nat Commun 2025; 16: 7244. 10.1038/s41467-025-62493-4 40770177 PMC12328812

[13] Clift PD . Controls on the erosion of Cenozoic Asia and the flux of clastic sediment to the ocean. Earth Planet Sci Lett 2006; 241: 571–80. 10.1016/j.epsl.2005.11.028

[14] Curray JR, Emmel FJ, Moore DG. The Bengal Fan: morphology, geometry, stratigraphy, history and processes. Mar Pet Geol 2003; 19: 1191–223. 10.1016/S0264-8172(03)00035-7

[15] Calvès G, Huuse M, Clift PD et al. Giant fossil mass wasting off the coast of West India: the Nataraja submarine slide. Earth Planet Sci Lett 2015; 432: 265–72. 10.1016/j.epsl.2015.10.022

[16] Gee MJR, Uy HS, Warren J et al. The Brunei slide: a giant submarine landslide on the North West Borneo Margin revealed by 3D seismic data. Mar Geol 2007; 246: 9–23. 10.1016/j.margeo.2007.07.009

[17] João HM, Badesab F, Gaikwad V et al. Controls of mass transport deposit and magnetic mineral diagenesis on the sediment magnetic record from the Bay of Bengal. Mar Pet Geol 2021; 128: 104994. 10.1016/j.marpetgeo.2021.104994

[18] Liang C, Liu CY, Xie XN et al. Basal shear zones of recurrent mass transport deposits serve as potential reservoirs for gas hydrates in the Central Canyon area, South China Sea. Mar Geol 2021; 441: 106631. 10.1016/j.margeo.2021.106631

[19] Cao L, Jiang T, Wang Z et al. Provenance of Upper Miocene sediments in the Yinggehai and Qiongdongnan basins, northwestern South China Sea: evidence from REE, heavy minerals and zircon U-Pb ages. Mar Geol 2015; 361: 136–46. 10.1016/j.margeo.2015.01.007

[20] He Y, Xie X, Kneller BC et al. Architecture and controlling factors of canyon fills on the shelf margin in the Qiongdongnan Basin, northern South China Sea. Mar Pet Geol 2013; 41: 264–76. 10.1016/j.marpetgeo.2012.03.002

[21] Zhao R, Chen S, Olariu C et al. A model for oblique accretion on the South China Sea margin; Red River (Song Hong) sediment transport into Qiongdongnan Basin since Upper Miocene. Mar Geol 2019; 416: 106001. 10.1016/j.margeo.2019.106001

[22] Chen HY . Establishment of Regional Stratigraphic Framework and Analysis of Hydrocarbon Accumulation in the Qiongdongnan Basin of Northern South China Sea. *Master Dissertation*, Ocean University of China, 2015.

[23] Li XB, Ge JW, Zhao XM et al. Geochemistry of Quaternary sediments in the northwestern South China Sea: sediment provenance and mid-Pleistocene transition. Mar Geol 2024; 477: 107371. 10.1016/j.margeo.2024.107371

[24] Kong F, Gao R, Gao SS et al. Mantle flow underneath the South China Sea revealed by seismic anisotropy. Natl Sci Rev 2023; 10: nwad176. 10.1093/nsr/nwad176 37671331 PMC10476890

[25] Milliman JD, Farnsworth KL. River Discharge to the Coastal Ocean: A Global Synthesis. Cambridge: Cambridge University Press, 2013.

[26] Tanabe S, Saito Y, Vu QL et al. Holocene evolution of the Song Hong (Red River) delta system, northern Vietnam. Sediment Geol 2006; 187: 29–61. 10.1016/j.sedgeo.2005.12.004

[27] Wan SM, Li AC, Clift PD et al. Development of the East Asian monsoon: mineralogical and sedimentologic records in the northern South China Sea since 20 Ma. Paleogeog Paleoclimatol Paleoecol 2007; 254: 561–82. 10.1016/j.palaeo.2007.07.009

[28] Sun Q, Maselli V, Wang X et al. Controls on canyon formation along mud-rich continental margins. Geology, in press. 10.1130/G54477.1

[29] Løseth H, Gading M, Wensaas L. Hydrocarbon leakage interpreted on seismic data. Mar Pet Geol 2009; 26: 1304–19. 10.1016/j.marpetgeo.2008.09.008

[30] Huhn K, Arroyo M, Cattaneo A et al. Modern submarine landslide complexes. In:Ogata, K, Festa, A, Pini, GA (eds.). Submarine Landslides: Subaqueous mass Transport Deposits from Outcrops to Seismic Profiles. Washington: American Geophysical Union and John Wiley & Sons, Inc., 2020, 183–200.

[31] Pauli CK, Ussler W, Dillon WP. Potential role of gas hydrate decomposition in generating submarine slope failures. In: Max MD (eds.). Natural Gas Hydrate. Coastal Systems and Continental Margins. Dordrecht: Springer, 2000.

[32] Urlaub M, Talling PJ, Masson DG. Timing and frequency of large submarine landslides: implications for understanding triggers and future geohazard. Quat Sci Rev 2013; 72: 63–82. 10.1016/j.quascirev.2013.04.020

[33] Ai F, Kuhlmann J, Huhn K et al. Submarine slope stability assessment of the central Mediterranean continental margin: the Gela Basin. In: Krastel S, Behrmann J, Völker D et al. Submarine Mass Movements and Their Consequences. Cham: Springer, 2014, 225–36.

[34] Flemings PB, Long H, Dugan B et al. IODP Expedition 308 Scientists, Pore pressure penetrometers document high overpressure near the seafloor where multiple submarine landslides have occurred on the continental slope, offshore Louisiana, Gulf of Mexico. Earth Planet Sci Lett 2008; 269: 309–25. 10.1016/j.epsl.2007.12.005

[35] Leynaud D, Mienert J, Vanneste M. Submarine mass movements on glaciated and non-glaciated European continental margins: a review of triggering mechanisms and preconditions to failure. Mar Pet Geol 2009; 26: 618–32. 10.1016/j.marpetgeo.2008.02.008

[36] Hoang VL, Clift PD, Schwab AM et al. Large-scale erosional response of SE Asia to monsoon evolution reconstructed from sedimentary records of the Song Hong-Yinggehai and Qiongdongnan basins, South China Sea. Geol Soc Lond Spec Publ 2010; 342: 219–44. 10.1144/SP342.13

[37] Clark MK, Schoenbohm LM, Royden LH et al. Surface uplift, tectonics, and erosion of eastern Tibet from large-scale drainage patterns. Tectonics 2004; 23: TC1006. 10.1029/2002TC001402

[38] Clift PD, Sun Z. The sedimentary and tectonic evolution of the Yinggehai-Song Hong basin and the southern Hainan margin, South China Sea: implications for Tibetan uplift and monsoon intensification. J Geophys Res Solid Earth 2006; 111: B06405. 10.1029/2005JB004048

[39] Farnsworth A, Lunt DJ, Robinson SA et al. Past East Asian monsoon evolution controlled by paleogeography, not CO_2_. Sci Adv 2019; 5: eaax1697. 10.1126/sciadv.aax1697 31692956 PMC6821471

[40] Métivier F, Gaudemer Y. Stability of output fluxes of large rivers in South and East Asia during the last 2 million years: implications on floodplain processes. Basin Res 1999; 11: 293–303. 10.1046/j.1365-2117.1999.00101.x

[41] Laberg JS, Vorren TO, Mienert J et al. Preconditions leading to the Holocene Trænadjupet Slide offshore Norway. In: Submarine Mass Movements and Their Consequences. Dordrecht: Kluwer Academic Publishers, 2003, 247–54.

[42] Gales JA, McKay RM, Santis LD et al. Climate-controlled submarine landslides on the Antarctic continental margin. Nat Commun 2023; 14: 2714. 10.1038/s41467-023-38240-y 37202379 PMC10195823

[43] Mienert J, Vanneste M, Bünz S et al. Ocean warming and gas hydrate stability on the mid-Norwegian margin at the Storegga Slide (Special Issue Ormen Lange). Mar Pet Geol 2005; 22: 233–44. 10.1016/j.marpetgeo.2004.10.018

[44] Beelen D, Wood LJ. Predicting bottom current deposition and erosion on the ocean floor. Basin Res 2023; 35: 1985–2009. 10.1111/bre.12788

[45] Sun QL, Cartwright JA, Xie XN et al. Reconstruction of repeated Quaternary slope failures in the northern South China Sea. Mar Geol 2018; 401: 17–35. 10.1016/j.margeo.2018.04.009

[46] Li W, Jing S, Urlaub M et al. Sea-level variations influence weak layer formation and submarine landslides on a low-latitude continental margin. Commun Earth Environ 2025; 6: 950. 10.1038/s43247-025-02949-z

[47] Chen FH, Xu QH, Chen JH et al. East Asian summer monsoon precipitation variability since the last deglaciation. Sci Rep 2015; 5: 11186. 10.1038/srep11186 26084560 PMC4471663

[48] Wan SM, Clift PD, Li AC et al. Tectonic and climatic controls on long-term silicate weathering in Asia since 5 Ma. Geophys Res Lett 2012; 39: L15611. 10.1029/2012GL052377

[49] Su M, Xie X, Xie Y et al. The segmentations and the significances of the Central Canyon System in the Qiongdongnan Basin, northern South China Sea. J Asian Earth Sci 2014; 79: 552–63. 10.1016/j.jseaes.2012.12.038

