# Supplementary material for: Basin uplift and related climate dynamics drive far-field submarine landslides
Source: Natl Sci Rev. 2026 Jun 4;13(13):nwag342. doi: 10.1093/nsr/nwag342 (PMC13348244; doi:10.1093/nsr/nwag342)
Supplement: nwag342_Supplemental_File [file nwag342_supplemental_file.docx]

**Supplementary file for**

Basin uplift and related climate dynamics drive far-field submarine landslides

Qiliang Sun^a,b*^, Vittorio Maselli^c^, Xingxing Wang^a^, Shucheng Xie^a^

^a^State Key Laboratory of Geomicrobiology and Environmental Changes, Hubei Key Laboratory of Marine Geological Resources, China University of Geosciences, Wuhan, Hubei, China

^b^Laboratory for Marine Mineral Resources, Qingdao National Laboratory for Marine Science and Technology, Qingdao 266061, China

^c^Department of Chemical and Geological Sciences, University of Modena and Reggio Emilia, Modena, Italy

Corresponding author: Qiliang Sun, [sunqiliang@cug.edu.cn](mailto:sunqiliang@cug.edu.cn), +86 18086030221

**Contents of this file**

Supplementary texts

Supplementary Figures S1 to S8

Supplementary References

Supplementary Text 1

**Calculation of sedimentation rates**

The volume of solid particles does not change during the sediment compaction, but the volume of voids between the solid particles is reduced during the compaction. Therefore, the volumes of solid particles before and after the depositional compaction could be expressed as [1]:

$\int_{y_{1}}^{y_{2}} \left[ 1-\varphi(y) \right]dy=\int_{y_{1}^{'}}^{y_{2}^{'}} \left[ 1-\varphi(y) \right]dy$ (1)

Where $\varphi(y)$ is the porosity at depth y; $y_{1}$ and $y_{2}$ are the top and base depths of a certain strata at present situation (burial); $y_{1}^{'}$ and $y_{2}^{'}$ are top and base depths of a certain strata before compaction.

In general, the porosity of sedimentary rocks changes exponentially with depth, which can be expressed as below:

$\varphi(y)=\varphi_{0}exp(-cy)$ (2)

Where $\varphi_{0}$ is the original porosity that is the porosity around the seabed, and c is the coefficient of compaction.

According to the Formula (1) and (2), we can get the decompaction formula that is expressed as: $y_{2}^{'}-y_{1}^{'}=y_{2}-y_{1}-\frac{\varphi_{0}}{c}\left[ \exp\left( -cy_{1} \right)-exp(-cy_{2}) \right]+\frac{\varphi_{0}}{c}\left[ \exp\left( -cy_{1}^{'} \right)-exp(-cy_{2}^{'}) \right]$ (3)

Where $y_{2}^{'}-y_{1}^{'}$ is the sediment thickness before compaction. Because the top of sediment column has been recovered to the seabed (the top of sediment column before compaction is zero), $y_{1}^{'}$ is equal to zero, and thus $y_{2}^{'}$ is equal to the strata thickness before compaction. To get $y_{2}^{'}$, we need to know the values of $y_{1}$，$y_{2}$, c and $\varphi_{0}$.


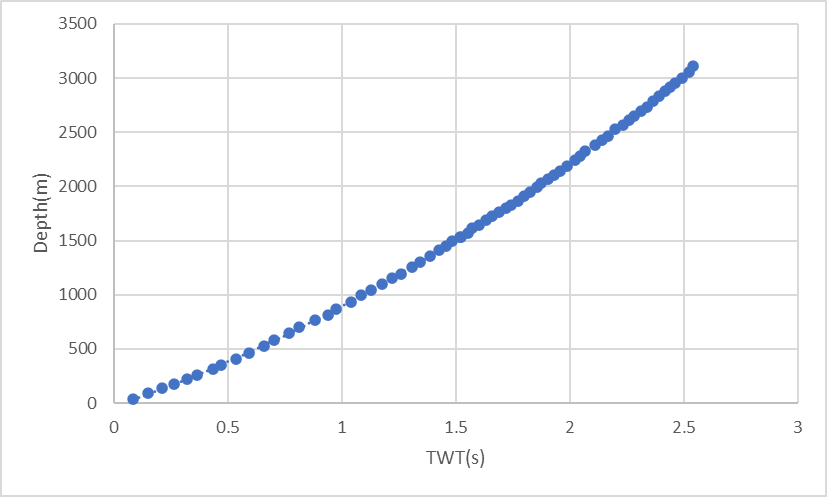


Figure 1: Time-depth conversion relationship in the study area (modified from [2]). TWT = Two way travel time

According to the results of Well A (Fig. 1; [2]), time-depth conversion relationship (goodness-of-fitting R^2^ of 0.9999) in the study area can be expressed as:

$y=195.89t^{2}+731.74t-33.402$ (4)

Where y is the burial depth (unit in m), and t is the two-way travel time (unit in s). According to the equation (4) and the measured top/base depth in two-way travel time on the seismic profiles, we can get the top/base depth in meter. The present-day tops and bases of five sequences of the underformed strata around the shelf-edge trajectories (pink dashed lines in Figures 2-3 in the main text) in the western and eastern seismic profiles are shown in Tables 1&2.

Table 1: Present-day top and base burial depths of sediment sequences from the western seismic profile

| Sequence | Top  (ms) | Base  (ms) | Base Horizon | Depth of top (s) | y1(m) | Depth of base (s) | y2 (m) |
| --- | --- | --- | --- | --- | --- | --- | --- |
| SU1 | 196.2 | 1273.43 | T20 | 0 | 0 | 1.08 | 982.16 |
| SU2 | 1133.61 | 1412.72 | T27 | 0.94 | 824.67 | 1.22 | 1146.68 |
| SU3 | 1237.09 | 1607.34 | T29 | 1.04 | 940.49 | 1.41 | 1389.26 |
| SU4 | 1482.39 | 1821.15 | T30 | 1.29 | 1231.81 | 1.62 | 1672.88 |
| SU5 | 1775.01 | 2155.37 | T40 | 1.58 | 1610.16 | 1.96 | 2152.09 |

Table 2: Present-day top and base burial depths of sediment sequences from the eastern seismic profile

| Sequence | Top  (ms) | Base  (ms) | Base Horizon | Depth of top (s) | y1(m) | Depth of base (s) | y2 (m) |
| --- | --- | --- | --- | --- | --- | --- | --- |
| SU1 | 291.96 | 1172.98 | T20 | 0 | 0 | 0.88 | 766.14 |
| SU2 | 1172.98 | 1414.59 | T27 | 0.88 | 766.14 | 1.13 | 1038.01 |
| SU3 | 1414.59 | 1684.26 | T29 | 1.13 | 1038.01 | 1.39 | 1368.47 |
| SU4 | 1741.01 | 2007.73 | T30 | 1.45 | 1441.64 | 1.72 | 1802.44 |
| SU5 | 2007.73 | 2318.15 | T40 | 1.72 | 1802.44 | 2.03 | 2257.44 |

The compaction coefficients of mudstone and sandstone are 1.18 and 0.45, respectively [3]. The well data shows that the Pliocene-Quaternary strata mainly comprise mudstone (90%), and thus their compaction coefficient (c) is ~1.107 (c = 1.18×0.9+0.45×0.1); whilst the late Miocene strata comprise more sandstone (~20%) that that (~10%) of the Pliocene-Quaternary strata, and thus the compaction coefficient is ~1.034. Moreover, the original porosity is ~65.73% inferred from [3].

According to the $y_{1}$，$y_{2}$, c and $\varphi_{0}$ that have been gotten from the above-mentioned calculation, we can calculate the $y_{2}^{'}$ from equation (3). The present-day strata thickness (after compaction) ($y_{2}-y_{1}$) and decompacted strata thickness ($y_{2}^{'}-y_{1}^{'}$) are listed in Tables 3&4.

Table 3: The present-day strata thickness and decompacted strata thickness of the western seismic profile

| Sequence | Base Horizon | y2-y1(m) | y2' -y1' (m) | Sedimentation rate (m/Myr) |
| --- | --- | --- | --- | --- |
| SU1 | T20 | 982.17 | 982.17 | 549 |
| SU2 | T27 | 322.00 | 504.71 | 561 |
| SU3 | T29 | 448.77 | 681.10 | 454 |
| SU4 | T30 | 441.07 | 703.72 | 541 |
| SU5 | T40 | 541.93 | 866.53 | 173 |

Table 4: The present-day strata thickness and decompacted strata thickness of the eastern seismic profile

| Sequence | Base Horizon | y2-y1(m) | y2' -y1' (m) | Sedimentation rate (m/Myr) |
| --- | --- | --- | --- | --- |
| SU1 | T20 | 766.14 | 766.14 | 426 |
| SU2 | T27 | 271.87 | 431.14 | 479 |
| SU3 | T29 | 330.46 | 540.00 | 360 |
| SU4 | T30 | 360.80 | 614.04 | 472 |
| SU5 | T40 | 455.01 | 765.68 | 153 |

Supplementary Text 2

**Calculation of excess pore pressure**

We use the Finite Element (FE) software ABAQUS to perform the seepage-consolidation analysis of saturated porous media, employing the u-p formulation as the governing equation to calculate the excess pore pressure:


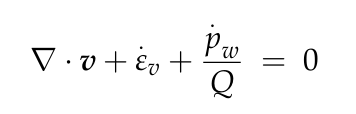


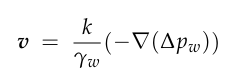


where *v* represents the fluid flow velocity; *ε_v_* represents the volume strain; *p_w_* represents the pore pressure; Δ*p_w_* represents the excess pore pressure; *k* represents the hydraulic conductivity, and *γ_w_* represents the bulk density of the pore fluid.


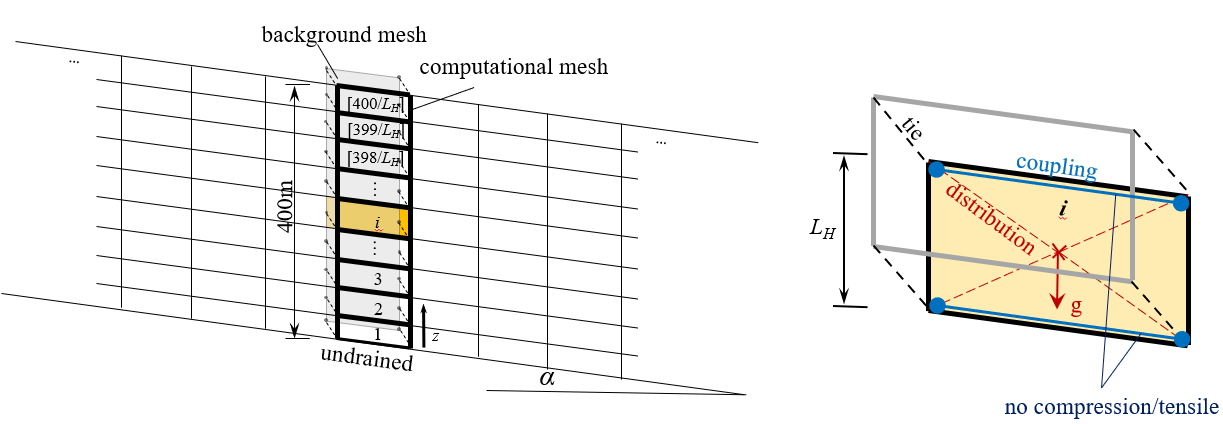


Figure 2: (a) Finite element numerical model for this study; (b) the load and boundary conditions of the i-th layer

In numerical implementation, the slope sedimentary process is simplified as an accumulation process on an inclined, rigid, and impermeable substrate (Fig. 2), and the model-change technique is employed to simulate the layer-by-layer accumulation of sediments. In each sedimentary increment, a CPE4P (4-node bilinear plane strain quadrilateral) layer coupled with pore pressure is activated above the existing grid. The consolidation time of this layer is determined by the ratio of activated layer thickness to sedimentation rate to ensure the temporal synchrony of the sedimentation-consolidation process. To avoid changes in the position of the top nodes of the newly activated layer caused by settlement and plastic deformation during the sedimentation process, a set of mesh replicas is specially introduced within the existing framework. This set of mesh replicas (hereafter referred to as the background mesh) shares an identical mesh structure with the computational slope seabed mesh (hereafter referred to as the computational mesh), but uses CPE4 elements without the pore pressure degree of freedom. The background mesh is modeled as a linear elastic material with extremely low elastic modulus and is completely detached from external loads and boundary constraints. A strain coupling relationship is established between the background mesh and the computational mesh through full-node tied contact. For detailed methodology, please refer to references [4-5].

Table 5: The sedimentation rate and the thickness of activated layer during the deposition process

| Periods | Sedimentation rates | Activation layer thickness/number of sedimentation layers | Consolidation time |
| --- | --- | --- | --- |
| 5500-4200kyr b.p. | 0.541 m/kyr | 700 m/70 | 18.484 kyr |
| 4200-2700kyr b.p. | 0.454 m/kyr | 680 m/68 | 22.02 kyr |
| 2700-1800kyr b.p. | 0.561 m/kyr | 500 m/50 | 17.82 kyr |
| 1800kyr b.p.--Present | 0.546 m/kyr | 980 m/98 | 18.31 kyr |

A one-dimensional infinite model is established to reproduce the sedimentation process and to quantitatively analyze the pore water pressure. The thickness of each layer is *L_H_* =10 m. The height of the activation layer and the consolidation time are shown in Table 5. The mechanical behavior of sediment compaction and consolidation is quantified by using the modified MCC model. The model assumes that during the sedimentation process, the initial pore ratio, the initial stress state, and the dry density parameters remain constant. Meanwhile, considering the dynamic process of permeability coefficient with pore ratio, the classical logarithmic relationship is adopted to describe the changes of hydraulic conductivity *k* with void ratio *e*.

Table 6: Parameters used in this study

| Parameters | Values(Units) |
| --- | --- |
| Slope inclination *α* | 6.2° |
| Initial void ratio *e*_0_ | 2.33 |
| Internal friction angle *φ'* | 30° |
| Initial hydraulic conductivity *k*_0_ | 5×10^-7^ m/s |
| Compression index *c_c_* | 0.6 |
| Swell index *c_s_* | 0.15 |
| Dry density of soil particles *ρ*_s_ | 2.7 g/cm^3^ |
| Initial stress-required layer height *h*_0_ | 0.3 m |
| Plasticity index *I_P_* | 35 |
| Relationship between permeability coefficient *k* and porosity *e* | $k=5\times10^{\frac{e-2.0}{0.4}-7}\text{m/s}$ |

The parameters used in this study are from the geophysical interpretation and collected from references [4-5]. The model parameters are shown in Table 6. To avoid the reconstruction influence of node deformation on the gravitational load during the deposition process, the continuous medium coupling method is adopted to apply the equivalent gravitational concentrated load to the centroid of the current sedimentary layer, and it is assigned to the nodes within the layer through the shape function. In terms of the boundary conditions, by constraining the rotational freedom degrees of all nodes and the translational freedom degrees of the corresponding nodes on both sides of the sedimentary cylinder through displacement coupling, we achieve the key condition of the infinite slope assumption (the strain rate d*εs*/d*t* = 0 along the slope and normal directions) (Fig. 2). At the top of the sedimentary layer, a dynamically changing hydraulic boundary is applied with the activation of deposition layer.


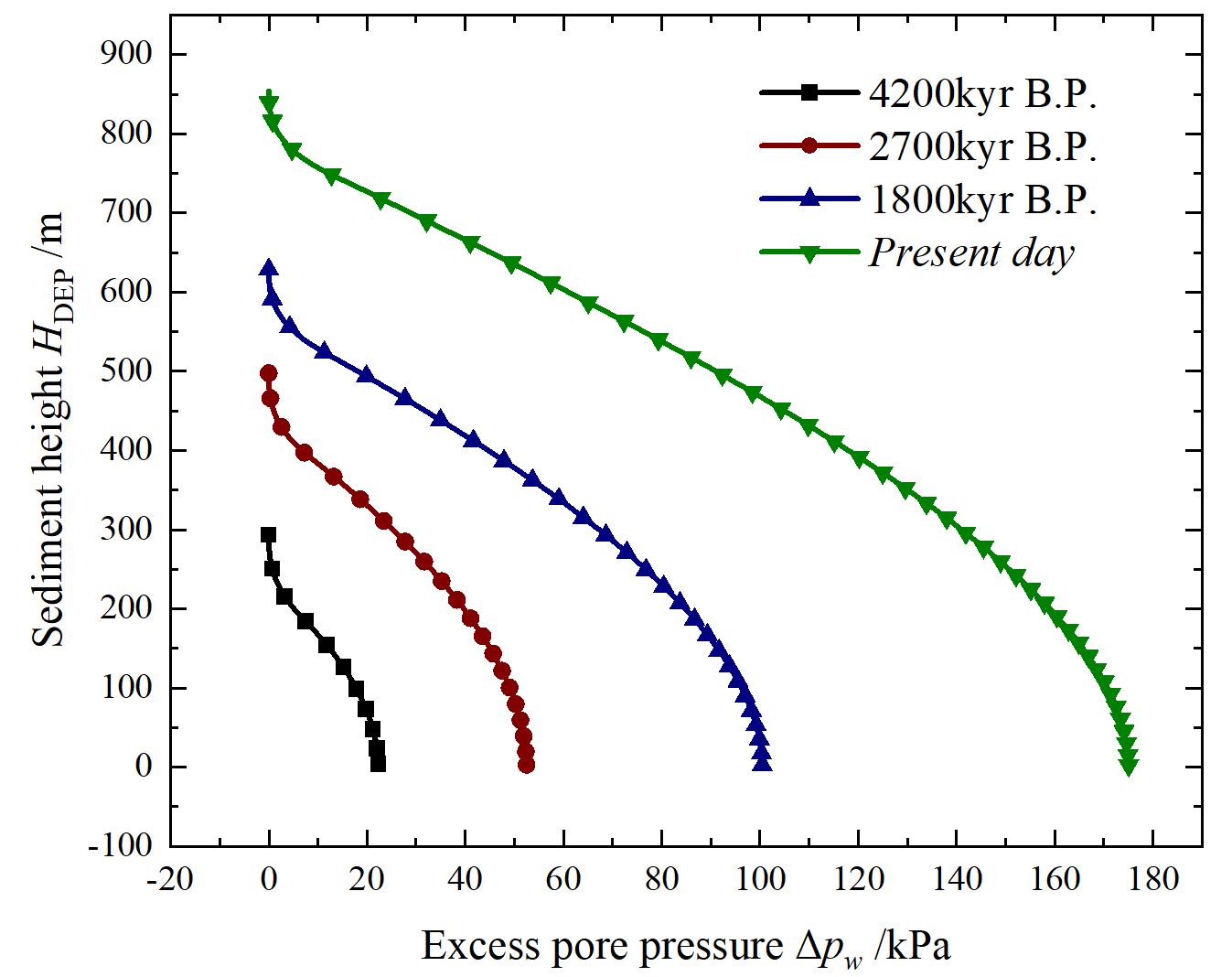


Figure 3: The evolution characteristics of excess pore pressure during the deposition process in four scenarios (4200 kyr B.P., 2700 kyr B.P., 1800 kyr B.P. and *Present day*)

The simulation result shows that the excess pore pressure is less important in the 0 - ~50 m depth and then quickly increases from ~50 m downwards (Fig. 2). In 4.2 Myr B.P. scenario, the excess pore pressure reached up to 20 kPa at the bottom of the deposition column; while it can reached up to 53 kPa in 2.7 Myr B.P scenario (Fig. 3).

Figure S1


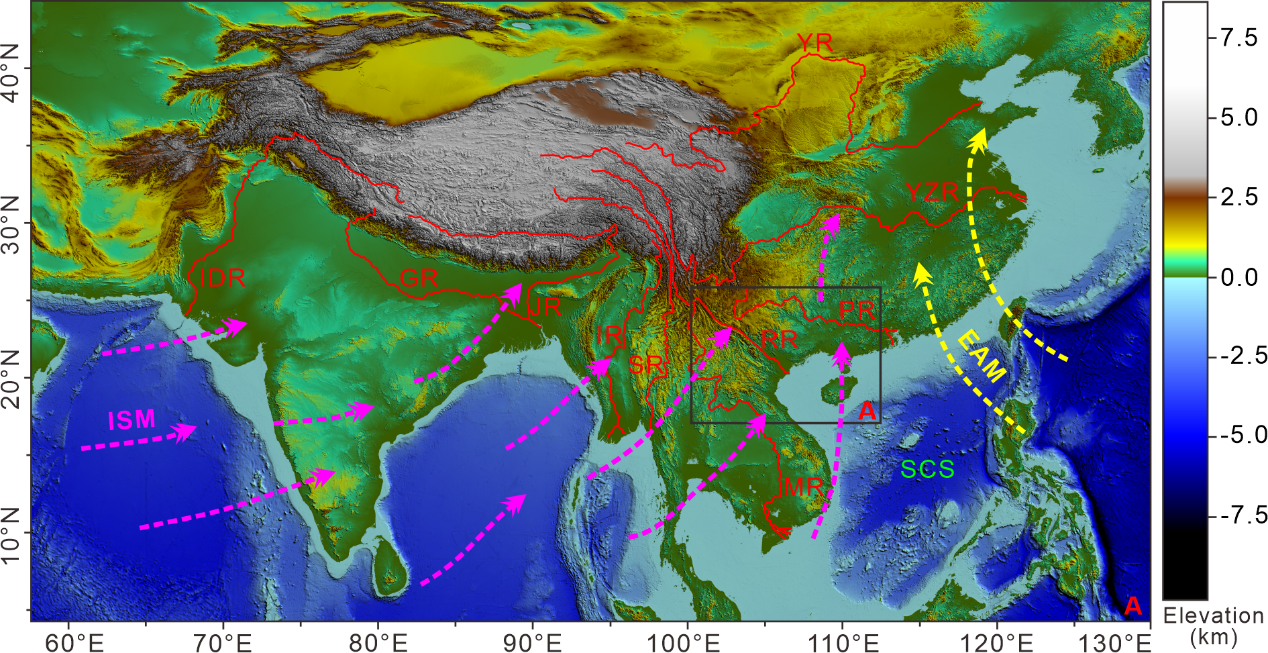


Figure S1: Geological settings of the study area. A: Regional topography of South and East Asia with highlighted monsoonal systems (modified from [6]), with the pink arrows representing the Indian Summer Monsoon (ISM) transporting moisture from the Arabian Sea and Bay of Bengal toward the Himalayas and the yellow arrows marking the East Asian Monsoon (EAM), which brings moisture from the western Pacific toward eastern China. The blue arrows indicate surface currents in the South China Sea (SCS). Several major rivers are outlined in red, including the Yellow River (YR), Yangtze River (YZR), Pearl River (PR), Red River (RR), Mekong River (MR), Salween River (SR), Irrawaddy River (IR), Jamuna River (JR), Ganges River (GR), Indus River (IDR). The black square outlines Figure 1A.

Figure S2


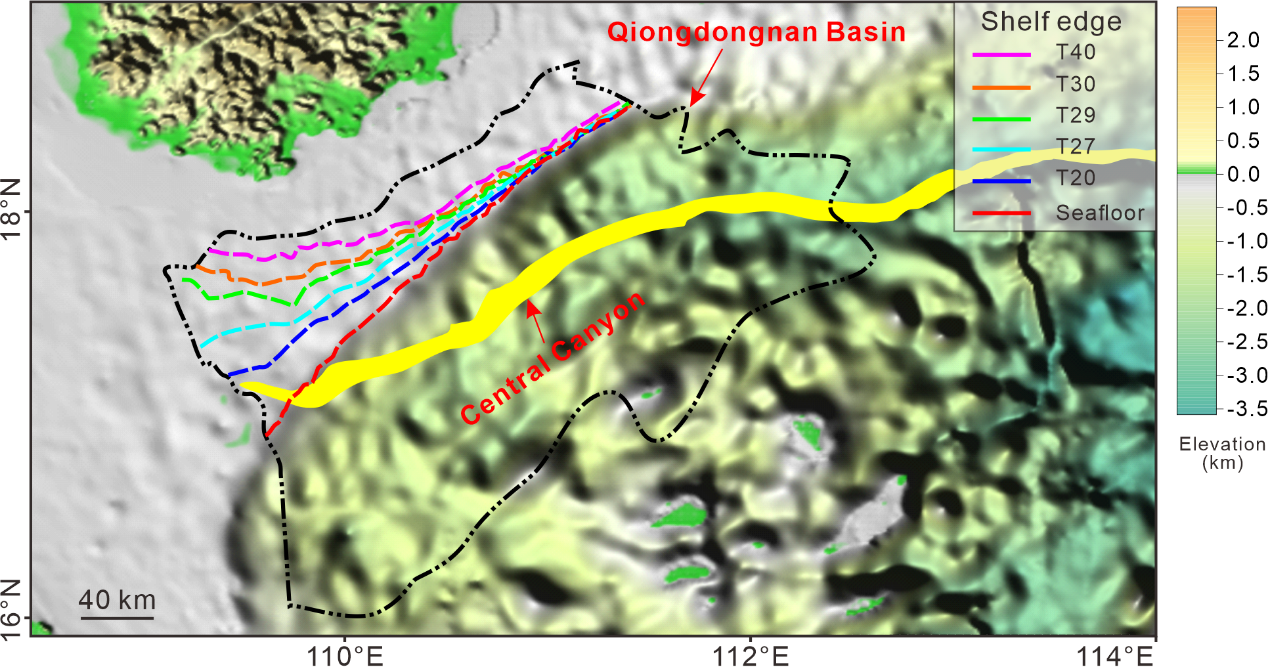


Figure S2: Enlargement of the Qiongdongnan Basin (black dashed line). The projections of shelf-edge trajectories of T40 (pink dashed line), T30 (brown dashed line), T29 (green dashed line), T27 (cyan dashed line), T20 (blue dashed line) and present seafloor (red dashed line) are marked on the present-day seabed. The extension of Central Canyon (yellow band) is also marked.

Figure S3


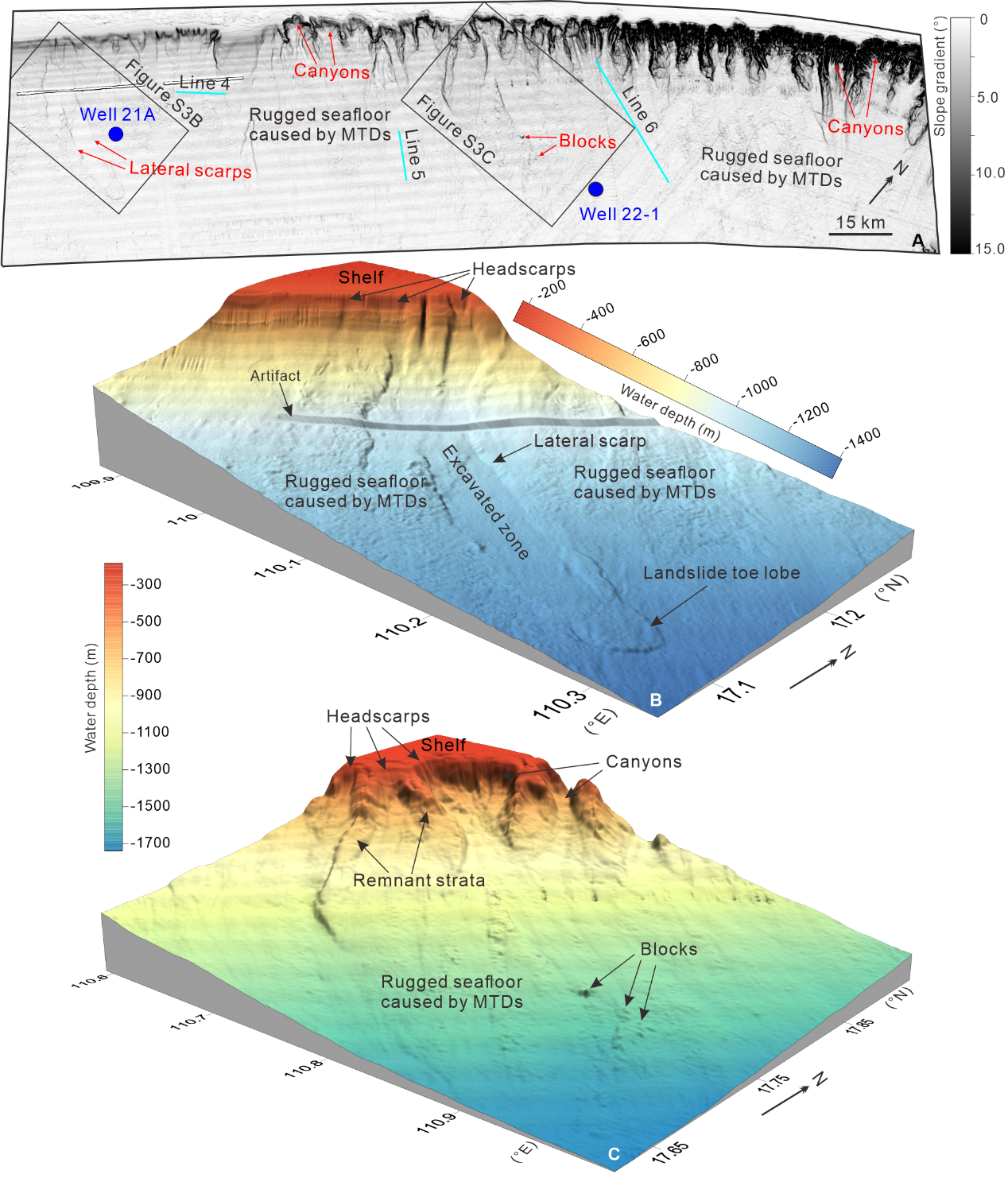


Figure S3: A: Slope gradient map showing canyons, slope failure scarps and landslide deposits (rugged seafloor) in the study area. Locations of Figures 5A (Line 4), 5B (Line 5), 5C (Line 6), Well 22A and Well 22-1 are labelled; B and C: seabed morphologies showing the headscarps/lateral scarps of mass-transport deposits (MTDs), depositional lobe, canyons, blocks and rugged seafloor.

Figure S4


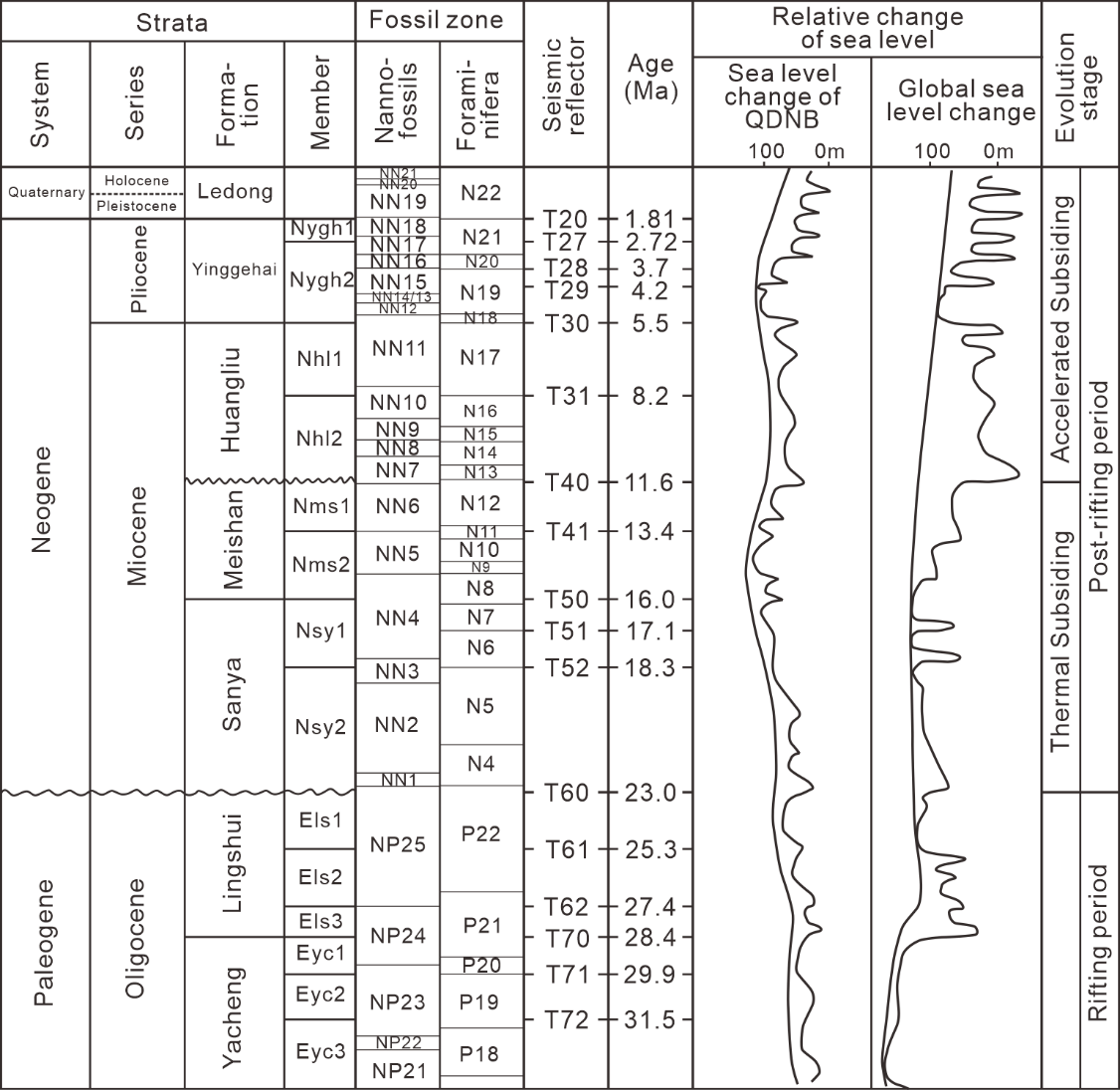


Figure S4: The sketch of sequences, fossil assemblages, sea level change curves and tectonic evolution stages of the Qiongdongnan Basin (modified from [7]). The fossil zones are adopted from [8] and the global sea level curve is from [9].

Figure S5


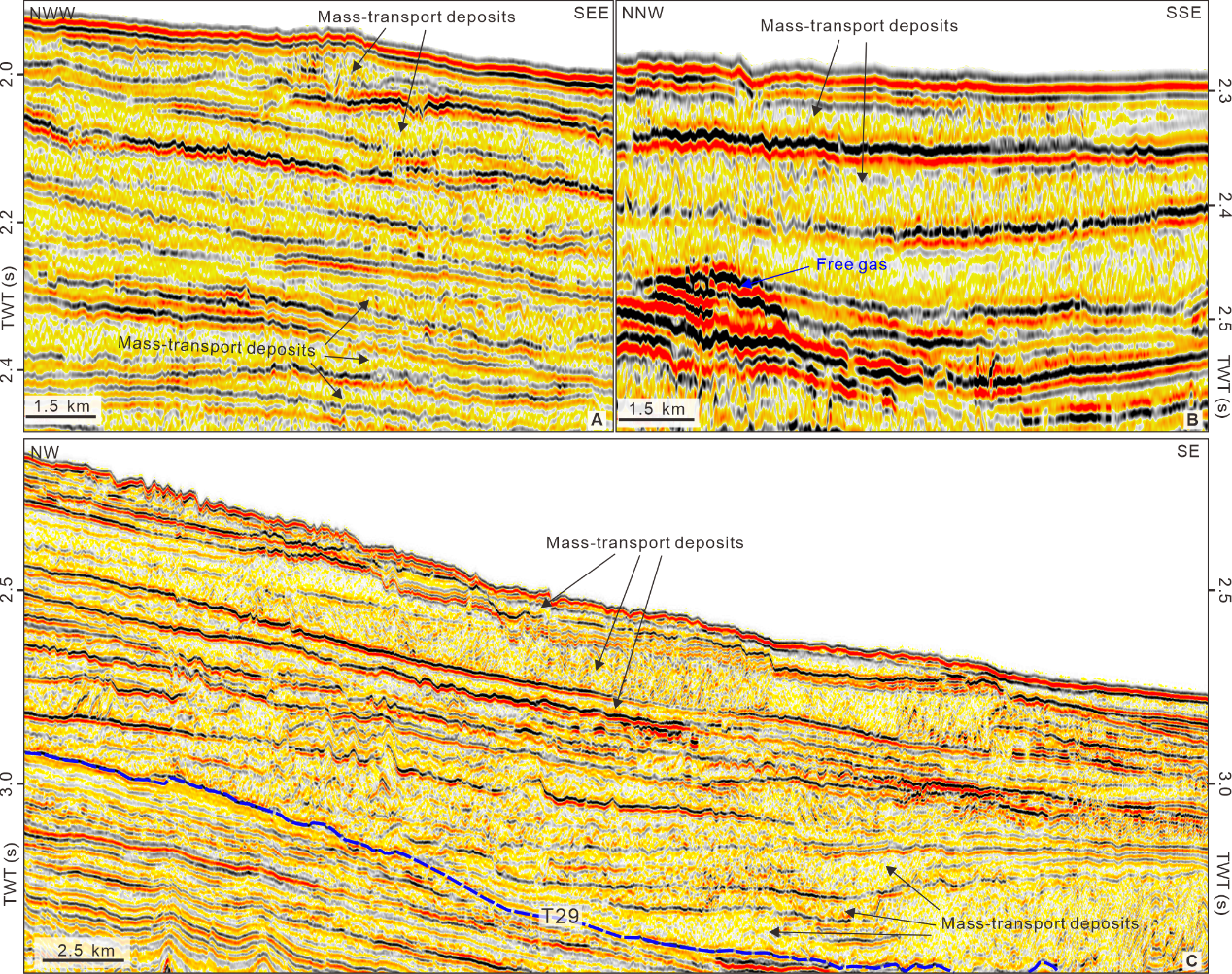


Figure S5: Seismic profiles (Lines 4, 5 and 6) showing the seismic characteristics of mass-transport depositions in the western (A), middle (B) and eastern (C) Qiongdongnan Basin. Mass-transport deposits are characterized by chaotic seismic reflections. Enhanced seismic anomalies (free gas) are also marked in (b). See locations in Figure 3A.

Figure S6


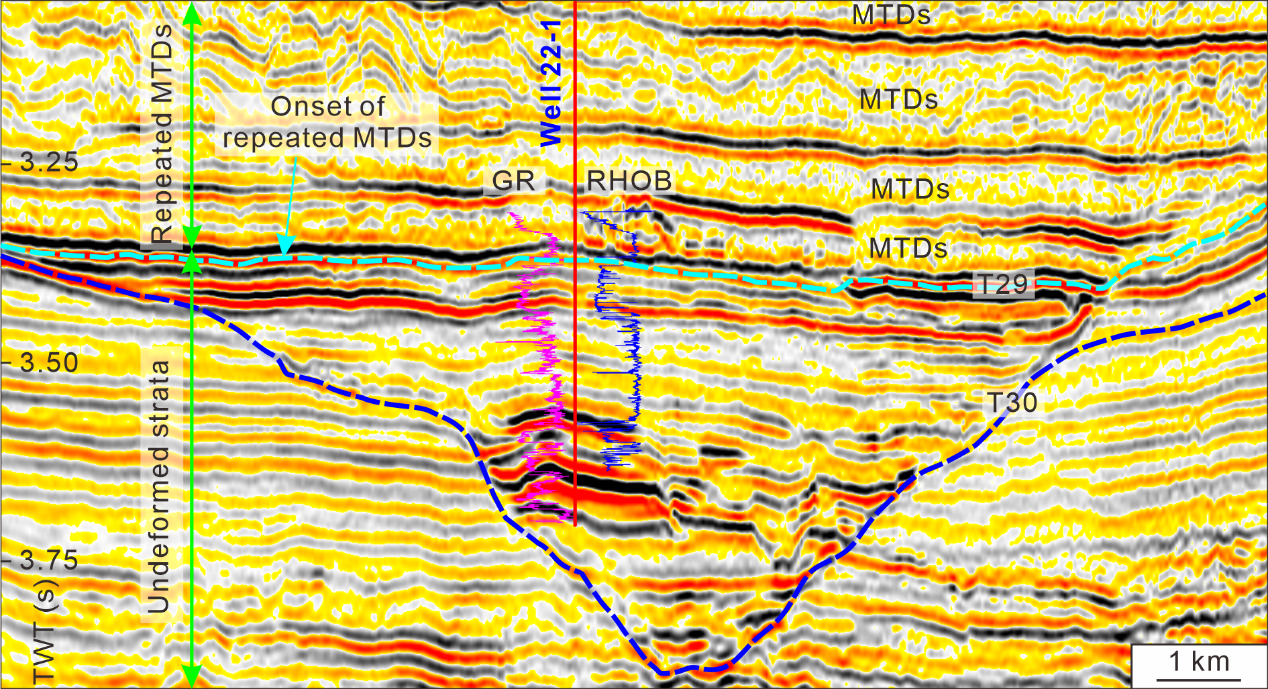


Figure S6: Well 22-1 drilled through the Central Canyon. Note that whilst the strata above T29 show chaotic seismic reflections with strong, negative boundaries representing stacked MTDs, the strata below T29 are characterized by sub-parallel reflections with variable amplitudes intercalated with transparent units likely associated to turbidite channel deposits.

Figure S7


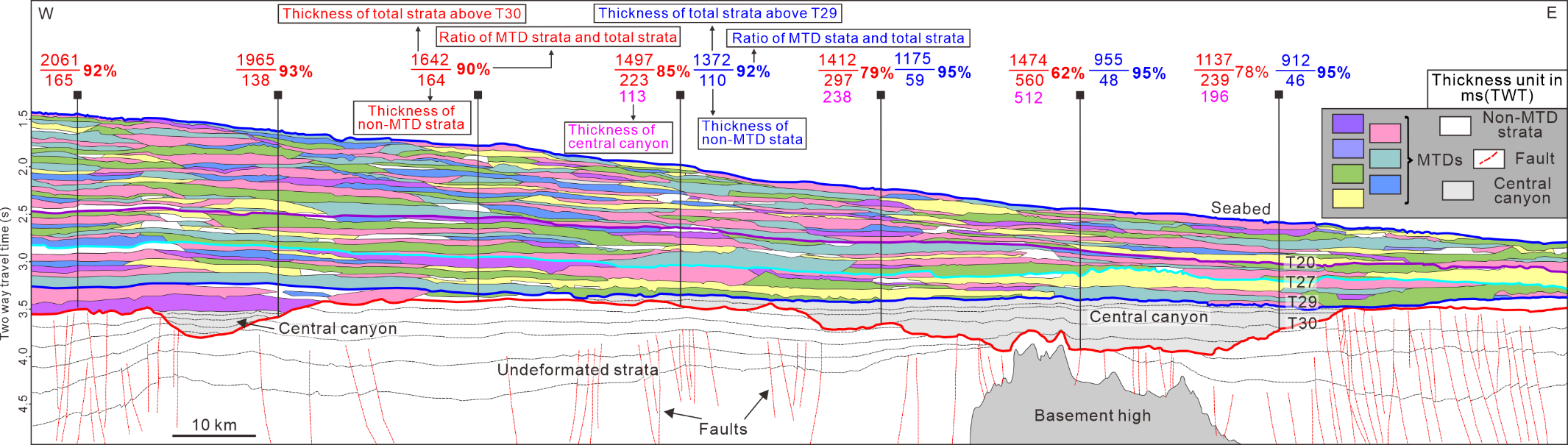


Figure S7: Selected seismic profile (modified from Figure 4B) used to calculate the approximate ratio of mass-transport deposits (MTDs) vs other slope deposits in the Pliocene and Quaternary succession. The red numbers are the thickness calculated from surface T30. The blue numbers are calculated from surface above T29. Pink number is the thickness of Central Canyon. In general, excluding the thickness of Central Canyon, >90% of the Pliocene and Quaternary strata comprise MTDs.

Figure S8


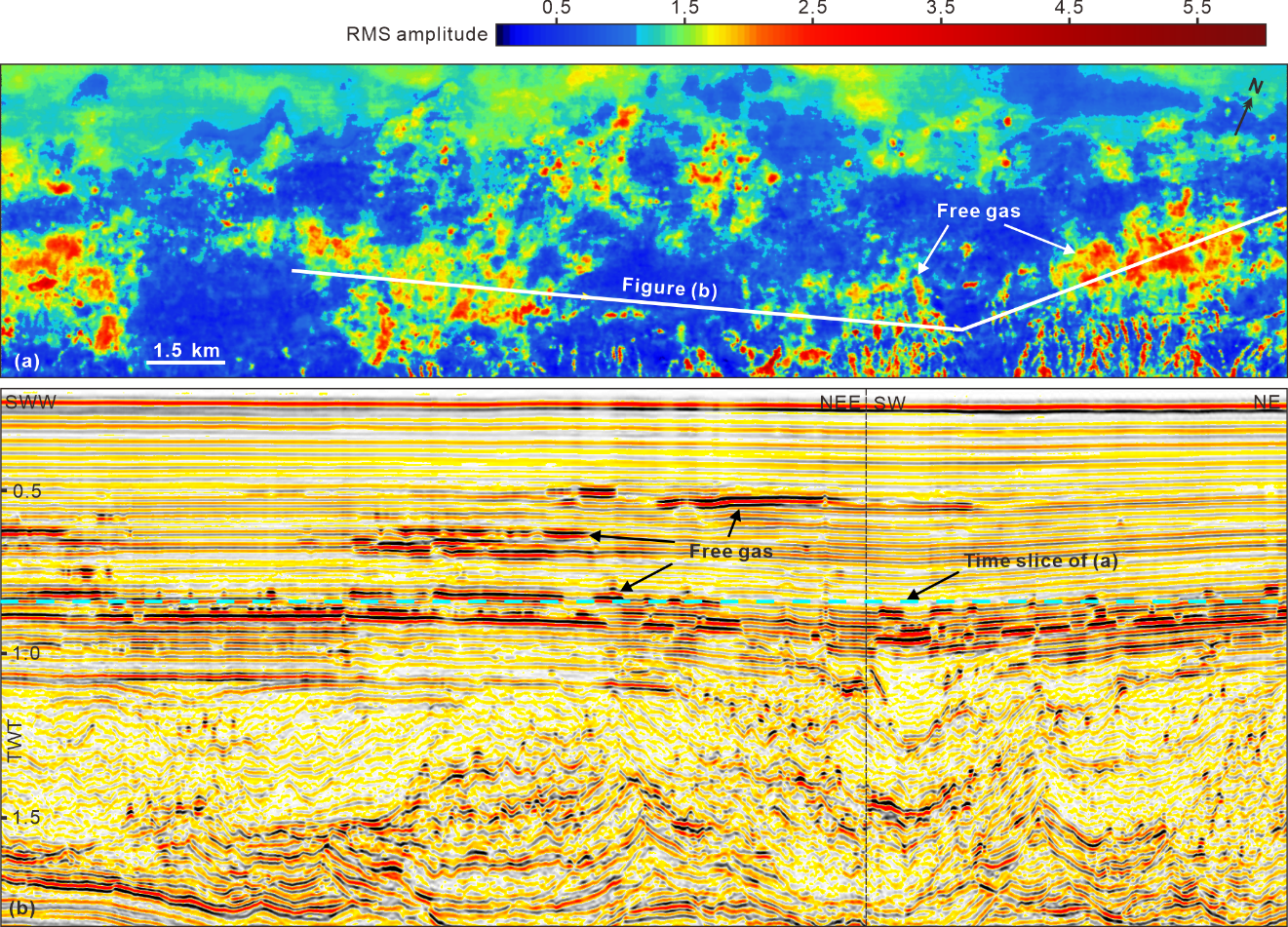


Figure S8: Seismic characteristics of free gas. (a) Time slice of RMS (root mean square) amplitude showing the distribution of free gas in map view (high value of RMS amplitude). Time window is 50 ms above and below the cyan dashed line; (b) seismic profile showing free gas as enhanced negative seismic anomalies.

**References**

1. Sclater, J.G., Christie, P.A.F., 1980 Continental stretching: An explanation of the PostMid-Cretaceous subsidence of the central North Sea Basin. *J. Geophys. Res.* 85, 3711-3739.

2. Liu, B., Zhou, J.X., Ren, K.Y., Liu, A.Q., 2014. The Time-Depth relationship of deviated wells in the slope-break belts of the Qiongdongnan Basin. *Natural Gas Industry* 34, 36-40.

3. Xie, H., Zhou, D., Shi, H.C., Li, Y.P., Kong, D.M., 2021. Comparative study on the Cenozoic tectonic and sedimentary evolution in the deep water areas of the Zhujiang River Estuary Basin and the Qiongdongnan Basin. *Haiyang Xuebao* 43, 48-61.

4. Wang, Z., Zheng, D., Gu, Z., Guo, X., Nian, T., 2014. A methodology to evaluate the real-time stability of submarine slopes under rapid sedimentation. *J. Mar. Sci. Eng.* 12, 823.

5. Stoecklin, A., Friedli, B., Puzrin, A.M., 2017. Sedimentation as a control for large submarine landslides: mechanical modeling and analysis of the Santa Barbara Basin. *J. Geophys. Res. Solid Earth* 122, 8645-8663.

6. Wen, X.Y., Liu, Z.Y., Wang, S.W., Cheng, J., Zhu, J., 2015. Correlation and anti-correlation of the East Asian summer and winter monsoons during the last 21,000 years. *Nat. Commun.* 7, 11999.

7. Su, M., Xie, X., Xie, Y., Wang, Z., Zhang, C., Jiang, T., He, Y., 2014. The segmentations and the significances of the Central Canyon System in the Qiongdongnan Basin, northern South China Sea. *J. Asian Earth Sci.* 79, 552-563.

8. Gong, Z.S., Li, S.T., 1997. Continental margin basin analysis and hydrocarbon accumulation of the northern South China Sea. *Science Press*, pp. 193-256.

9. Haq, B.U., Hardenbol, J., Vail, P.R., 1987. Chronology of ﬂuctuating sea-levels since the Triassic. *Science* 235, 1156-1167.
